# Influence of Graphene Oxide Contents on Mechanical Behavior of Polyurethane Composites Fabricated with Different Diisocyanates

**DOI:** 10.3390/polym13030444

**Published:** 2021-01-30

**Authors:** Nadia Akram, Muhammad Saeed, Muhammad Usman, Asim Mansha, Fozia Anjum, Khalid Mahmood Zia, Irfan Mahmood, Nida Mumtaz, Waheed Gul Khan

**Affiliations:** 1 Department of Chemistry, Government College University Faisalabad, Faisalabad 38000, Pakistan; msaeed@gcuf.edu.pk (M.S.); musman@gcuf.edu.pk (M.U.); mansha.asim@gmail.com (A.M.); foziaanjum2008@yahoo.com (F.A.); drkmzia@gcuf.edu.pk (K.M.Z.); irfanmehmood5703@gmail.com (I.M.); razamoona135@gmail.com (N.M.); 2 Department of Chemistry, Quaid-i-Azam University, Islamabad 45320, Pakistan; jadoon_gul@yahoo.com

**Keywords:** polyurethane, graphene oxide, FTIR, TGA, DMA, SEM

## Abstract

The exceptional behavior of graphene has not yet been entirely implicit in the polymer matrix. To explore this fact in the present work, two series of Polyurethan (PU) composites were synthesized. The structural modification was observed by the use of two different diisocyanate of methylene diisocyanate (MDI) and hexamethylene diisocyanate (HMDI) in hydroxylterminated polybutadiene (HTPB) by using I,4 Butane diol (BD) as the chain extender. The variation in hard segment up to 25 (wt.%) in both series led to significant changes in the mechanical behavior of graphene oxide (GO) induced composites. Both series were prepared by an in situ polymerization process. Fourier transform infrared (FTIR) analysis showed a peak in the region of 1700 cm^−1^, which confirmed the conversion of the NCO group into urethane linkages. Thermal gravimetric analysis (TGA) revealed a thermal stability up to 450 °C @ 90% weight loss. The swelling behavior showed the optimum uptake of 30% of water and 40% of dimethyl sulfoxide (DMSO) with aliphatic diisocyanate. The values of storage modulus (*E′*), complex modulus (E*), and compliance complex (D*) were observed up to 7 MPa, 8 Mpa, and 0.7 MPa^−1^, respectively. The degree of entanglement (*N*) values were calculated from DMA and were found in the range of 1.7 × 10^−4^ (mol/m^3^). Phase segregation of PU was observed by scanning electron microscopy (SEM), elucidating the morphology of composites.

## 1. Introduction

Two dimensional graphene and its derivatives have emerged as multipurpose fillers that can be used for the composites. They play a consequential part in the development of the structural, physicochemical, and mechanical properties of various polymers in the presence of several reactive functionalities such as epoxy, carboxyl, carbonyl, and hydroxyl groups [1,2]. Undoubtedly, the recently developed graphene based materials have become a new sensation in composite science and technology. The exceptional properties are not only constrained to graphene, but it is also transformed into derivative compounds such as graphene oxide and, reduced graphene oxide, etc. [3,4,5]. Graphene has the potential to gain thermal stability and can offer a large surface area with excellent mechanical strength to the matrix. This behavior makes it one of most powerful reinforcing agents for the fabrication of composites [6,7]. 

The specific requirements of the product entail great care in choosing the appropriate material. The sp^2^ hybridized carbon of graphene layers represents the exceptional Young’s modulus of one *Tera Pascal (TPa)*, which contains approximately 200 times greater breaking strength compared to steel. This has encouraged the exploration of this material’s mechanical strength by inducing as a filler in polymers [8,9,10]. The fast growing interest of researchers has developed numerous fabrication techniques for graphene-based polymer composites including melt mixing, solvent assisted casting/coating methods, extrusion techniques and electrospinning, etc. [11,12]. Despite several advantages offered by graphene and its derivative as a filler, the aggregation of graphene induces phase separation in the matrix, which relentlessly discourages the particle dispersion and deteriorates the properties of composites, particularly in the presence of graphene oxide (GO) as the reinforcing agent [13,14,15].

Thermoplastic polyurethane is one of the most versatile polymers, and is constituted by three monomers; the long chain diols are usually based on polyester or polyether linkages that are known as macrodiol/polyols, constituting the soft segments (SS) of the polymerized polyurethanes (PUs) [16]. The second most important part of polyurethane is the diisocyanate, which may be aliphatic, cycloaliphatic, or aromatic in nature, and the third monomer of this group is the chain extender, which may be an oligomer with diol or diamine functionalities. The combination of the diisocyanate and chain extender together develops hard segment (HS) contents. Both HS and SS are responsible for phase segregation of its architecture [17,18]. Together, the chemistry of all these constituents decide the nature and behavior of PUs. The HS is also marked for the polarity of PU due to the presence of the urethane group, while the SS constitutes the non-polar region in the PU architecture. Under such diverse conditions, the addition of a reinforcement helps to improve the thermo-mechanical and morphological behavior of PU [19,20].

The ardent concern of investigators in graphene oxide (GO) as a filler has always urged the need to explore diverse combination of polymers. Ramezanzadeh et al. [21] used modified GO sheets to prepare polyisocyanate-functionalized graphene oxide (PI–GO) at various functionalization reaction times of 24, 48, and 72 h. Ji et al. [22] prepared highly conductive composites by using silver nanowires (Ag–NWs) and a small amount of GO (0.25–0.33% relative to Ag-NWs). The reinforcement was dispersed in PU to observe the effect in composites. Bera et al. [23] investigated the effect of amine functionalized graphene oxide on the physico-mechanical properties of glycidol-terminated polyurethane (GPU) nanocomposites. Fan et al. [24] functionalized a composite filler of graphene oxide and functionalized nanocellulose that was synthesized by using isocyanatopropyltriethoxysilane (IPTS) as a coupling agent through a sol-gel process. They merged the filler into a waterborne polyurethane (WPU) dispersion to prepare the WPU composites.

The keen interest of the researchers to advent new fillers/ reinforcing agents is thriving. However, it is equally important to induce these reinforcing agents to utilize their full potential. GO is one of the fillers assumed to show inferior mechanical properties, but a better conductivity material. However, all of its mechanical aspects are actually under-covered. A small amount of information is available on the mechanical behavior of this exciting reinforcing agent in numerous polymers including PU. The lack of research, especially on the thermo-mechanical and morphological aspects, urges a dire need to fulfill this deficiency. Hence, the current work is a complete illustration of the thermo-mechanical behavior of PU composites reinforced with GO. The novelty of this work is the utilization of the vibrant chemistry of PU coupled with GO. The article aims to provide the complete structural, thermal, mechanical, and morphological information about the synthesized material. It will certainly aid researchers in finding a better matrix for capitalizing on its unique behavior. In this connection, two series of polyurethane composites were synthesized using GO as the reinforcing agent. In both series, HTPB was used as the macrodiol, the two different diisocyanates MDI and HMDI were used in each series, respectively, while 1,4 butane diol (BD) was used as the chain extender in both series. In order to develop the linear structure of the polymers, the NCO and OH ratio was maintained at one, while the GO quantity h varied from 0.5 to 2.0% in all compositions as fillers. The in situ polymerization method was adopted to polymerize PU samples. All the synthesized samples were subjected to detailed characterization including FTIR, TGA, DMA, and SEM to develop a clear and concise understanding of GO induced PU composites.

## 2. Materials and Methods

### 2.1. Materials

The targeted synthesis was carried out by using the following materials: hydroxyl terminated poly butadiene (HTPB *M_n_* = 3000 g−mol^−1^), (NESCOM, Islamabad, Pakistan) and 1,4 butane diol (BD), (Sigma Aldrich, St. Louis, MO, USA) were dried for 1 h in a vacuum oven at 80 °C. 4,4′-Methylenebis(phenyl isocyanate) (MDI) (Sigma Aldrich) and 4,4′-Methylenebis(cyclohexyl isocyanate) HMDI (Sigma Aldrich, St. Louis, MO, USA) were used without further purification. Graphene oxide powder (GO) (Sigma Aldrich, St. Louis, MO, USA), dimethyl sulfoxide (DMSO) (Sigma Aldrich), and acetone were used as solvents.

### 2.2. Fabrication of Polyurethane Composites

**Figure 1:**
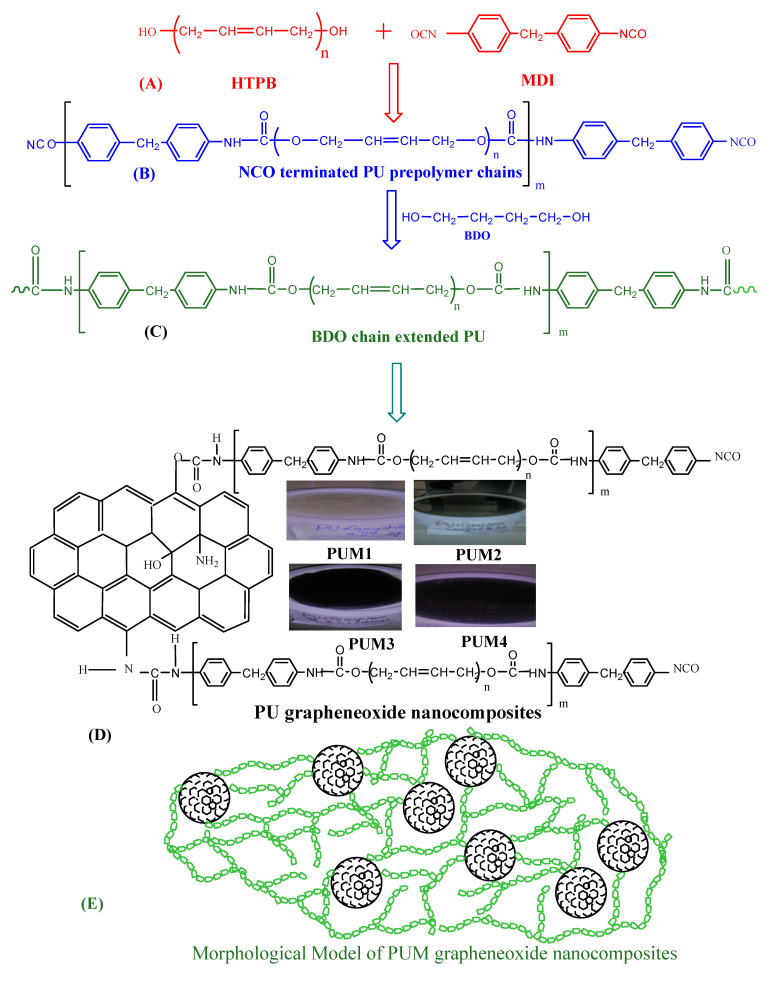

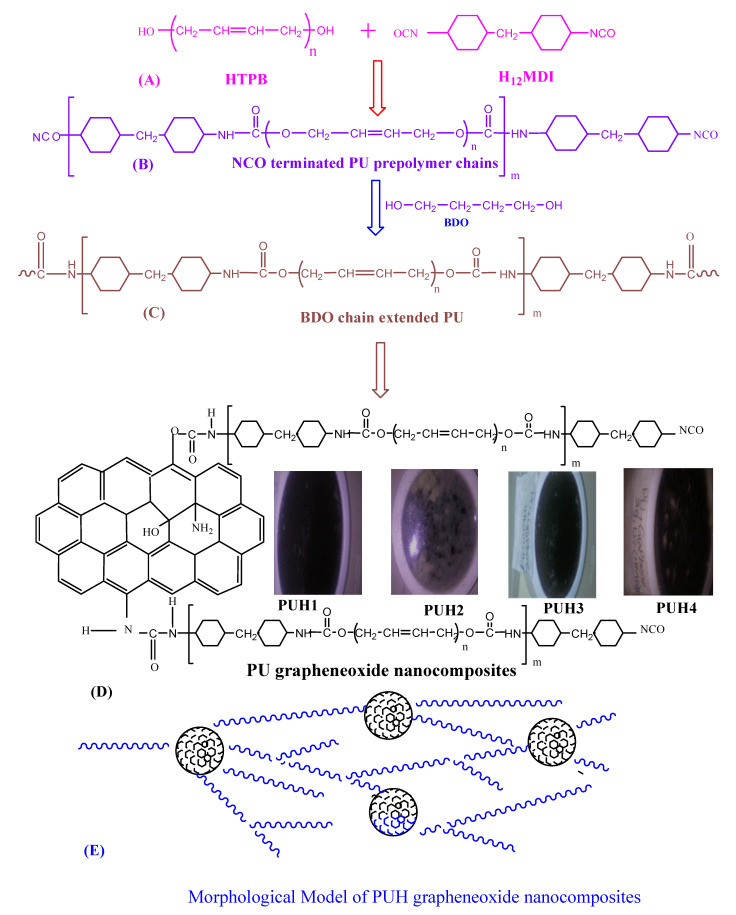
Synthetic route for the development of polyurethane and composites of the PUM and PUH series. (**A**) Addition of monomers (**B**) Formation of NCO terminated PU pre polymer chains (**C**) Polyurethane chain ex-tension with BD (**D**) Formation of graphene oxide nanocomposites (**E**) Morphology of graphene oxide induced nanocomposites.

The in situ polymerization process was utilized for the fabrication of polyurethane polymers as described in detail in our previous publications [25,26,27], while a reinforcing agent was added to synthesize the composites. The first step of polymerization was carried out by using a four necked round bottom flask fitted with a high speed mechanical stirrer, a thermometer, a reflux condenser, and N_2_ gas supply. The reaction was maintained in a nitrogen atmosphere of 10–12 bubbles/min in the pyrogallol solution. An oil bath was used to maintain the reaction temperature for the entire process. For the fabrication of polymers, the required amount of diisocyanate was used and the reaction temperature was increased up to 60 °C. The required quantity of dried macrodiol HTPB was added in the diisocyanate drop wise, increasing the temperature up to 100 °C. The continuous stirring for one hour developed a PU prepolymer with NCO terminal groups. The next step was proceeded, with vigorous stirring in the presence of BD, which converted the prepolymers into the final product. Chain termination reaction was proceeded for 15 min to obtain homogeneity in the reaction mixture by using 20 wt% of acetone. Finally, the temperature of the reaction mixture was reduced to attain the polymerized polyurethane as a blank sample. The composites were prepared by the addition of a calculated quantity of graphene oxide. The concentrations of the reinforcing agent were used in the range of 0.5 to 2 (wt.%) while maintaining the hard segment contents up to 25 (wt.%). The filler (GO) was added according to the optimized conditions of synthesis including temperature, stirring speed, and atmosphere of the synthesis. The reaction was carried out under a nitrogen atmosphere whereas the flow rate of N_2_ gas was maintained at the rate of 10–12 bubbles/min passing through the pyrogallol solution. In order to achieve the homogenous mixing of GO, vigorous stirring (1300 rpm) was maintained using a mechanical stirrer. The prepared samples were cast in polytetrafluoroethylene (PTFE) molds. The synthetic routes for the fabrication of two series of samples is provided in Figure 1 By degassing under vacuum at 50 °C for 8 h, the solvent in the cast samples evaporated. Stepwise drying of the film was performed in an oven at different temperatures for different time intervals. Formerly, the film was dried at 70 °C for 36 h, followed by drying at 110 °C for 4 h. The dried PU films were obtained in the thickness of 2 ± 0.05 mm and were stored in a desiccator at ambient temperature for further analysis. By following the procedure, two series of composites were prepared containing five samples in each series including a blank sample of the polymer without the addition of a reinforcing agent. Comprehensive detail about the stoichiometry of the samples is provided in Table 1. All the synthesized samples were subjected to structural analysis using FTIR, the thermal behavior was studied using TGA, the mechanical analysis was carried out using DMA, and the morphological analysis was performed using SEM. The water and DMSO retention tendency was observed using the swelling test.

### 2.3. Characterization

The synthesized samples were characterized using the following techniques;

#### 2.3.1. Fourier Transform Infrared Spectroscopy (FTIR)

The confirmation of the functional groups was carried out by recording spectra on a Fourier transform infrared spectrometer (FTIR, NICOLET 6700 spectrometer, Waltham, MA, USA). It was equipped with attenuated total reflectance, which was used as an adjunct. The spectra were recorded in the range of 4000–400 cm^−1^ using transmission mode.

#### 2.3.2. Thermal Gravimetric Analysis (TGA)

The analysis was executed on a Perkin Elmer (Waltham, MA, USA) thermogravimetric analyzer (TGA). The ramp rate of heating for the analysis was selected as 10 °C min^−1^ in the nitrogen atmosphere with a flow rate of 50 mL/min. The temperature range for thermograms was selected from 50 °C to 700 °C.

#### 2.3.3. Dynamic Mechanical Analysis (DMA)

The samples for DMA were prepared in polytetrafluoroethylene (PTFE) molds in the dimension of 50 mm in length, 2.5 mm in width, and 1–2 ± 0.5 mm in thickness. DMA (Q800; TA Instruments, New Castle, DE, USA) in temperature sweep mode was used to record the values of the storage modulus (*E′*), complex modulus (E*), and compliance complex (D*).

#### 2.3.4. Swelling Test

The polyurethane composites were immersed in distilled water and DMSO to evaluate their weight change, unless a constant weight was observed. Equation (1) was used to calculate %age of water/DMSO intake by the samples.

(1) $$\%\;\mathrm{water}/\mathrm{DMSO}\;\mathrm{absorption}=\frac{\mathrm{wet}\;\mathrm{weight}-\mathrm{Dry}\;\mathrm{weight}}{\mathrm{Total}\;\mathrm{weight}}\times 100$$

#### 2.3.5. Scanning Electron Microscopy (SEM)

The surface morphology was performed using a JEOL JSM-7000F, California, USA, with an accelerating voltage of 20 kV and a distance of 9.0 ± 0.5 mm.

## 3. Results

### 3.1. Structural Confirmation through FTIR

**Figure 2:**
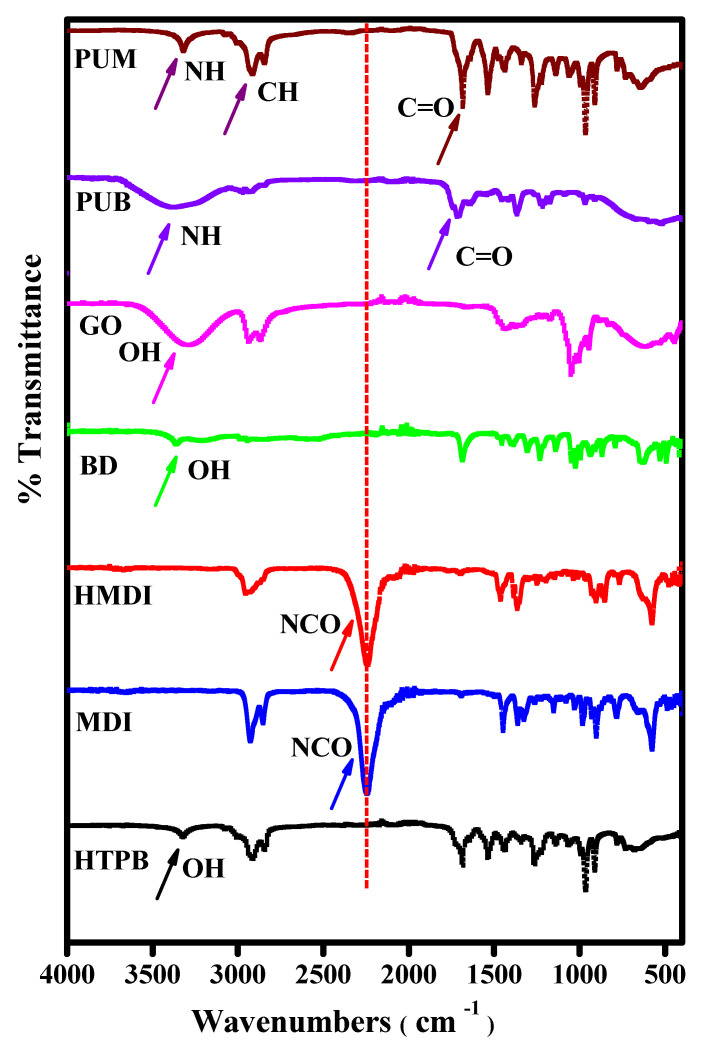
Fourier transform infrared (FTIR) spectra of monomers, polymer, and composite: HTPB (macrodiol), MDI and HMDI (diisocyanates), BD (chain extender), GO (reinforcing agent), PUB (blank polyurethane), and PUM (polyurethane composite).

All the monomers including HTPB, MDI, HMDI, BD, and GO, polyurethane blank, and GO reinforced composites were subjected to FTIR analyses. Table 2 indicates some characteristic peaks of amide linkage, carbonyl, symmetric and asymmetric vinyl linkages obtained from FTIR for the PU composites. The information indicates the presence of two absorption peaks as observed from the values; the presence of these values in the medium frequency area at 1615 cm^−1^ and 1720 cm^−1^ is a clear indication of stretching mode vibration of C=C and C=O of carboxylic acid and carbonyls functional groups correspondingly, which were located at the ends of graphene oxide. The FTIR spectra of all the monomers, PU, and PU composite induced with graphene oxide is presented in Figure 2. The peak at 1148 cm^−1^ indicated the stretching of the C–OH functional group offered by alcohol and a peak at 1028 cm^−1^ indicated the presence of C–O–C linkage in epoxide. The oxygen containing groups ensures the oxidation of graphite.

### 3.2. Evaluation of Thermal Stability by TGA

**Figure 3:**
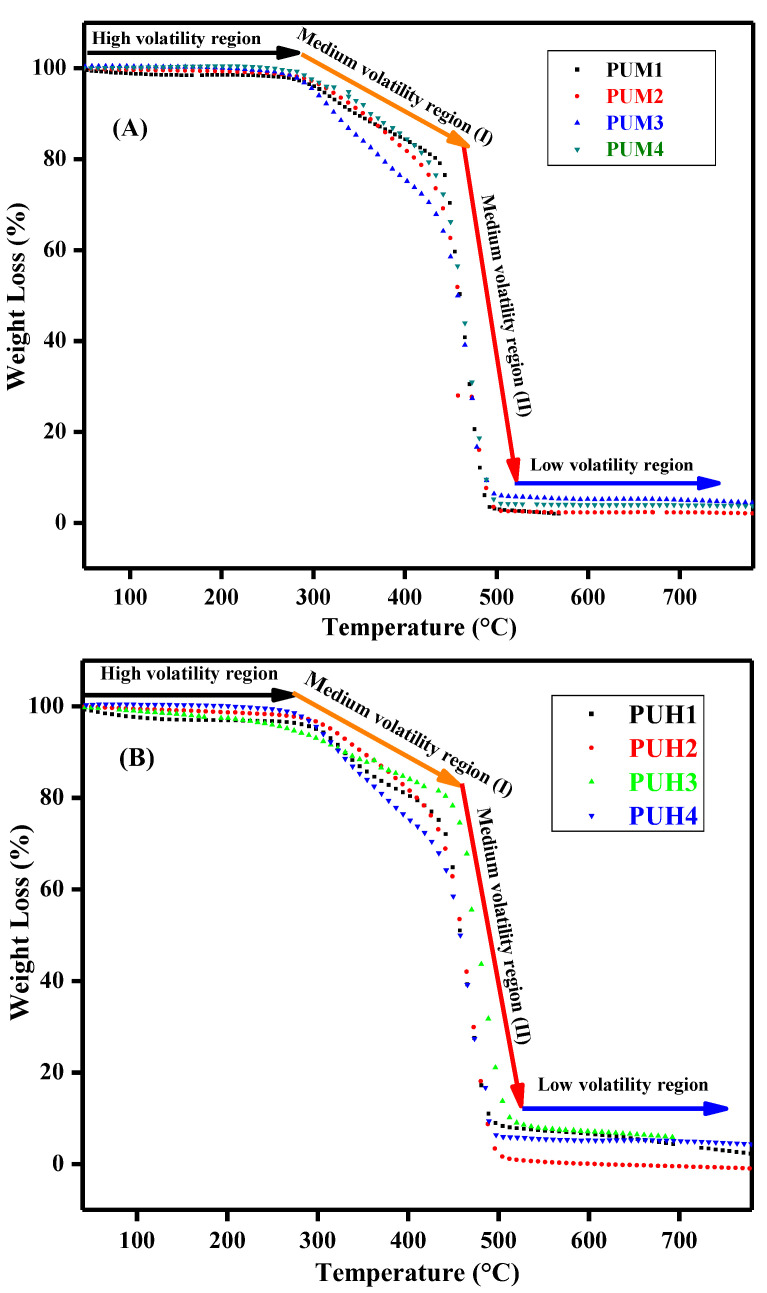
Thermalgravimetric analysis (TGA) thermograms of graphene oxide (GO) induced polyurethane (PU) composites. (**A**) Representation of TGA curves of PUM series. (**B**) Representation of TGA curves of PUH series.

Thermalgravimetric analysis (TGA) is always a reliable technique to measure the change in mass of the material with the function of temperature or time. Table 3 presents the data of weight loss (%) of the PU composites. The TGA thermograms of both series are given in Figure 3. The curves indicate that all the composites have the same pattern of decomposition, indicating an even dispersion of the HS and GO as filler in SS. It has also indicated the stability of the composites, which was found approximately up to 600 °C. As the initial stage of decomposition is actually a region of high volatility, which usually never decomposes, the actual sample leads to the low volatility region where the secondary and the primary bonds break down. There is a persistent decomposition of the composites in the region of medium volatility, which has ultimately decomposed the composites. For the PUM series up to 282 °C, a high volatility region was observed whereas the maximum stability was observed up to 490 °C. While in the (PUH) series, approximately, the same trend was observed with slight variation in the temperature, which is quite obvious with an approximately similar chemistry of the composites. Nevertheless, the central region of the curves, which is marked as the region of medium volatility is actually divided into two parts in both series. The first part was designated as stage I, which was a region of glassy to leathery state, whereas stage II appeared as a region of rubbery state. The multistage TGA curves of the PU composites were not exceptional as the architecture of this polymer is not based on a single monomer, rather it shows the combined effect of multiple components. Hence, the PU composites rarely show a perfect Tg according to composition and a slight variation can be observed. Apart from this, the thermal stability profile of PU composites was in a good range up to 500 °C. It is also a clear indication that the thermal stability of PU composites could be improved by the careful selection of the raw materials and the reinforcing agents. The trend of TGA was also correlated with the morphology of the samples where the even distribution of the samples was observed.

### 3.3. Mechanical Behavior of GO Induced PU Composites

**Figure 4:**
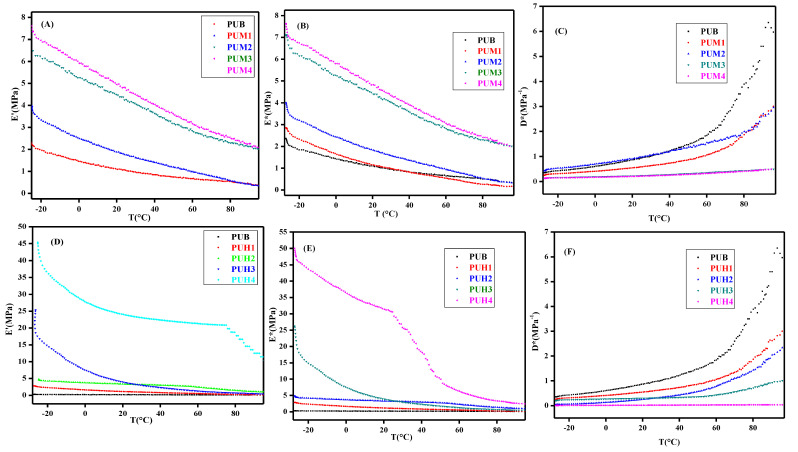
Mechanical Behavior of GO induced PU composites for PUM and PUH series. (**A**) Relationship of storage modulus and temperature of PUM. (**B**) Relationship of storage modulus and temperature of PUM complex modulus. (**C**) Relationship of compliance complex and temperature of PUM. (**D**) Relationship of storage modulus and temperature of PUH. (**E**) Relationship of complex modulus and temperature of PUH. (**F**) Relationship of compliance complex and temperature of PUH.

DMA is a very versatile technique that can be utilized for a complete mechanical profile for the polymers and composites. Table 4 provides a very detailed profile about the mechanical behavior of polyurethane composites, providing the values of storage modulus (*E′*), complex modulus (E*), complex compliance (D*), and degree of chain entanglements (N), which were calculated using the information of storage modulus. All these parameters proved significant to understand the behavior of composites, especially when it was reinforced with different concentrations of reinforcing agent. The value of (*E′*) provides an insight of reserved elastic energy, a slight change in the composition can be tracked with the help of *E′*. The significance of *E′* becomes eminent while comparing the compositional effect, which is primarily due to the interfacial adhesion between the matrix and the reinforcing agent. Figure 4 shows the temperature dependence of the *E′* on different GO filled composites. The *E′* values showed a gradual decrease with the temperature in both series (PUM and PUH), which is quite obvious due to the relaxation of the chains continuously. Occasionally, this change is sharp, depending on the HS and GO contents. The (*E′*) signifies the stiffness of the material as well as the molecular relaxation taking place as a function of temperature. The samples containing aromatic diisocyanates displayed higher values of *E′* compared to aliphatic diisocyanates, which is in accordance with the chemistry of aliphatic and cycloaliphatic compounds. However, as the stoichiometry was adjusted for both series, hence, a slight change was observed in both series. The *E′* was not higher, as expected for the composites and the most logical view is the contribution of soft segments, which was in excess, providing a soft flexible back bone to the polymers. There was also nominal variation in the blank PU and its composites filled with the lowest contents of GO, which however, increased gradually. The addition of fillers in the composites provided a large number of the functionalized group and developed strong bonding between the polymer matrices and cleaved the bond easily with temperature, which was the most probable reason of decrement in *E′* as the temperature increased.

A good dispersion of filler was observed in the matrix, indicated by higher values of *E′*. The Tg of PU is −76 °C [28], hence, usually there is a slight change in Tg with the addition of fillers. Figure 4B,B * represents the dependence of the dynamics modulus, also known as the complex modulus (E*), which is a viscoelastic property of the polymers and composites and a common method to describe the rubber belt hysteresis. It is also elaborated as a ratio of stress to strain under vibratory conditions. The representation of E* is elaborated in Equation (2), which is a combination of a real part elastic modulus *E′* and an imaginary part the loss modulus E″. The values of E* is also an indication of the stiffness of the material.

(2) $$E\;*=E^{\prime }+iE{}^{\prime \prime }$$

The values of the E* represent the trend given by the storage modulus. The GO, as a filler, has a positive impact on the values of E* with aromatic diisocyanate, hence, it also endorsed the results of the *E′*. The viscoelastic behavior of the samples was also evaluated by complex compliance (D*) for all the samples of the composites. The E* is considered as the complex modulus (relaxation in frequency domain) and D* is considered as the complex compliance (creep compliance in frequency domain) [29,30]. The viscoelastic trend clearly exhibited a change in the strength of the sample with temperature and it also indicated the slight variation in the mechanical behavior of the polyurethane composites with the small quantity of GO. It also predicted that the slight quantity of GO stabilized the PU composites, which are enough to be utilized in specific applications; however, the major changes could only be induced by increasing the HS contents. The lower mechanical strength can also be explained by the large contribution of the SS provided by HTPB. In order to understand the behavior of HTPB as SS, the degree of entanglement density (*N*) was calculated by using the expression as indicated by Equation (3). Entanglement develops the reticular structure, which may be in the coiled or globular structure. This is developed by the cross-linking points in the polymer chain or matrix of composites. This is a very important parameter as it decides the chain movement and greatly influences the nature of the polymer. It may also even restrict the normal movement of the polymer chains. It is one of the influential parameters of the rheology of polymers, which significantly plays its role. The factor was evaluated by DMA measurements using *E′*, while R is the universal gas constant and T is the absolute temperature.

(3) $$N=\frac{E{}^{\prime }}{6RT}$$

Table 4 shows very small values of *N*, which indicated the presence of low crosslink density and hence, the molecular chains were not entangled enough to display a high stiffness, even in the presence of a reinforcing agent. It also indicated that there was actually no chemical bonding involved between the filler and the matrix and it was supported by the physical cross linking where the filler was dispersed in the matrix.

### 3.4. Swelling Behavior of GO Induced PU Composites

**Figure 5:**
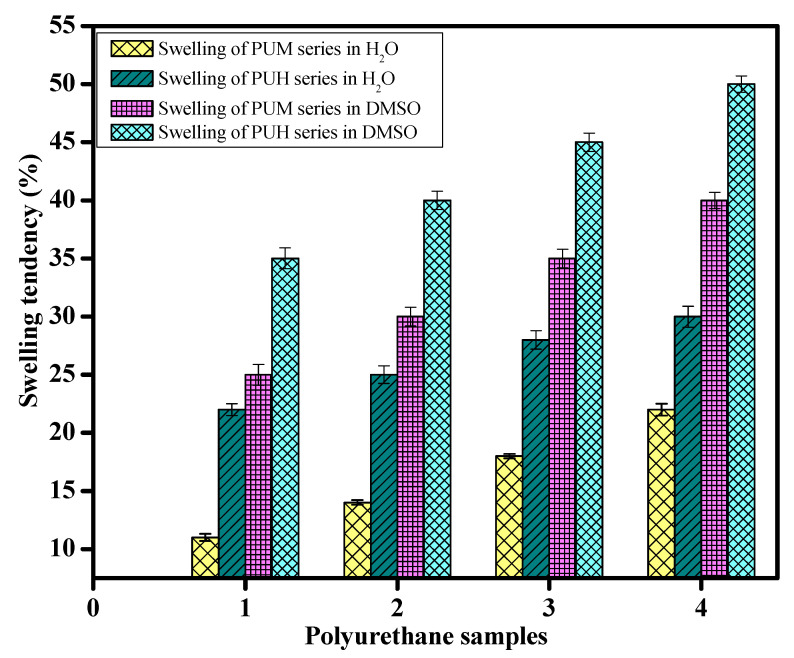
The swelling tendency of GO induced PU composites of the PUM and PUH series.

The penetration of solvent in the polymers is always a complex phenomenon, which has been tried to be understood on statistical grounds, yet it is very important as the interaction of the solvent cannot be avoided in the materials. As water is considered the universal solvent, it is worthy to at least find the behavior of composites toward water. In addition to this, there are numerous organic solvents, which completely or partially dissolve the polymer networks. Dimethyl sulfoxide (DMSO) is an important polar aprotic solvent, which has the ability to dissolves both polar and nonpolar compounds and is also miscible in a wide range of organic solvents even in water. It is always also considered a good choice to dissolve PU. Figure 5 represents the swelling behavior of both series in water and DMSO. This indicated that the samples based on aliphatic diisocyanates showed greater water absorption tendency compared to the PUM series. The most obvious reason was the crosslinking induced by the aromatic components, which hindered the penetration of the solvent. Moreover, the lower water penetration also indicated the greater contents of SS, which was comprised of nonpolar components. This behavior represents the phase segregation in the PU composites, which is more evident in the presence of GO. However, the swelling behaviour of the PU samples in DMSO is actually a complex phenomenon; the samples showed a significant increase in swelling compared to water, which is obvious due to the nature of DMSO. The careful observations indicated that DMSO showed a maximum absorption of up to 50%. This is greater % age of swelling than water. However, the higher swelling rate is not only difficult to detect, there can also be chances of a steady decline in swelling. The most probable reason is, after absorbing a certain quantity of solvent, the secondary bonding started to break, which actually deformed the structure of PU composites. It has a clear indication that the swelling of polymers can only be estimated under such circumstances, as the original weight of the subjected sample would no longer be sustained in the solvent; instead of retaining the structure of the sample, it would start dissolving in the solvents.

### 3.5. Morphology of GO Induced PU Composites by SEM

**Figure 6:**
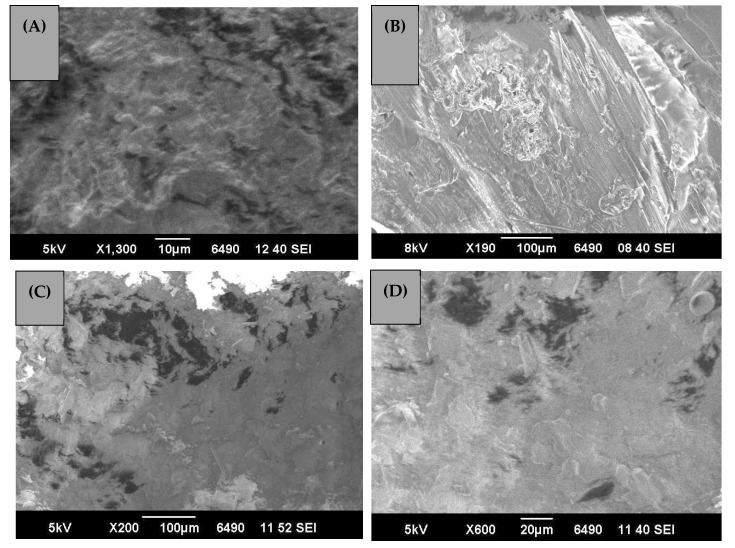
Scanning electron micrograph (SEM) of the GO-PU composites. (**A**) 10 μm scan of PUM4. (**B**) 100 μm scan of PUM4. (**C**) 100 μm scan of PUH4. (**D**) 10 μm scan of PUH4.

Figure 6A–D illustrates a SEM micrograph of various PU composites with GO as reinforcing agent, which showed flat or sheet like structures throughout the morphological images. Some areas of the surfaces were also covered with the crinkles, giving it an impression of coarse or uneven surface, bearing some defects in the structure. The micrograph displayed clear embedding of the reinforcement of GO in the PU matrix. The micrographs also showed a very low concentration of entanglements of polymer chains in the micrograph. Hence, all the components were nicely and evenly distributed without bulging at a particular center. Figure 6D also displays some transparent regions in the micrograph, while some areas appeared, giving the impression of a wave; this trend provides a hint of the presence of graphene in monolayers. All of the figures show that the GO remained embedded in various samples approximately equal at different magnifications, as presented in Figure 6A–D. These samples show the strong hydrogen bonding with PU chains, which is due to the reinforcing agent surrounded by the PU matrix. Figure 6B presents the encapsulation of GO in the matrix offered by PU in this case. Due to the thick coating of GO, the interactions between PU and GO is significantly evident. The presence of graphene planes is clearly seen and marked in Figure 6B. The micrographs actually exhibited roughness, and the edges and basal planes indicated the attachment of oxygen in GO. The available defected sites of GO developed strong bonding with oxygen. The composites indicated a more uniform dispersion of the fillers of GO regardless of the content of the fillers. The samples were homogeneous, at least at the microscopic scale, with the GO.

## 4. Conclusions

The hegemony of polyurethane in the vast polymer domain is not accidental, and the innumerable properties and tendency to be molded with numerous raw materials has specified its position among polymers. As the stoichiometry is the critical factor to evaluate the desired properties, two series of PU composites were synthesized, the matrix consisted of hydroxyl terminated polybutadiene (HTPB) as macrodiol, while the diisocyanate in both series were varied in nature. The diisocyanates were HMDI and MDI, keeping the constant chain extender of 1,4 butane diol (BDO) using graphene oxide (GO) as the reinforcing agent in the range of 0.5 to 2.5 (wt.%). The FTIR analysis confirmed the formation of the urethane linkage by showing the disappearance of the NCO peak and formation of the amide peak. The thermal stability observed by TGA was found to be up to 500 °C for both series with only a slight variation in different stages. The water penetration was observed by the swelling test, which showed 10 to 30% water absorption with the change in composition. The aromatic diisocyanates restricted the water penetration compared to the aliphatic diisiocyanates. The greater contents of SS also hindered the water penetration due to its non-polar nature. The greater swelling tendency of PU composites were observed in DMSO (up to 50%) compared to water. The DMA analysis showed consistency in the behavior of storage modulus (*E′*) up to 7 MPa, complex modulus (E*) up to 8 Mpa, and compliance complex up 0.7 MPa^−1^. The degree of entanglement (*N*) values were found in the very lower range. A clear segregation was observed, which indicated the typical behavior of PU samples. The quest to find the right composition of PU to utilize the optimum potential of GO has a promising future yet to be explored.

## Figures and Tables

**Table 1:** A comprehensive description of sample codes and stoichiometry of polyurethane composites containing aromatic/cycloaliphatic diisocyanate.

Sample Code	Graphene Oxide (wt.%)	HS Contents ^a^ (wt.%)	SS Contents ^b^ (wt %)	Sample Code	Graphene Oxide (wt.%)	HS Contents ^a^ (wt.%)	SS Contents ^b^ (wt. %)
PUB	0	5.0	95.0	PUB	0	5.0	95.0
PUM1	0.5	10.0	90.0	PUH1	0.5	10.0	90.0
PUM2	1.0	15.0	85.0	PUH2	1.0	15.0	85.0
PUM3	1.5	20.0	80.0	PUH3	1.5	20.0	80.0
PUM4	2.0	25.0	75.0	PUH4	2.0	25.0	75.0

In the composition of the PUM and PUB series, the NCO:OH ratio was maintained as 1. a = Hard Segment Content^25^ = [(W_MDI/HMDI_ + W_BD_ + W_Gr_)/W_Total_] × 100. b = Soft Segment Content = [100 − Hard Segment Content (%)].

**Table 2:** Wave numbers (cm^–1^) assignment of Fourier transform infrared (FTIR) spectra of polyurethane.

Assignments	Wave Number (cm^−1^)	
	Observed Values	Literature Values [16,17]
υsymCH_2_ = (*Vinyl*)	2978	2977
υasCH_2_	2935–2913	2935–2915
υsymCH_2_	2855	2865–2845
H–C=O	1716–1700	1750–1680
υC=O	1736–1709	1750–1680
υC–C(aromatic)	1599–1615	1615–1580
δN–H	1531–1529	1650–1550
υC–N	1231–1220	1250–1150
δC–H (aromatic)	1224–963	1225–950
υC–O–C	1060–1049	1150–1050
ω C–H (aromatic)	814–675	900–670

**Table 3:** Weight loss (%) data of polyurethane (PU) composites obtained by thermalgravimetric analysis (TGA).

Sample Code	T@1%Wt Loss (°C)	T@50%Wt Loss (°C)	T@90%Wt Loss (°C)	Sample Code	T@1%Wt Loss (°C)	T@50%Wt Loss (°C)	T@90%Wt Loss (°C)
PUM1	200	457	488	PUH1	120	457	489
PUM2	242	457	488	PUH2	135	460	488
PUM3	257	459	481	PUH3	160	470	489
PUM4	282	457	490	PUH4	258	457	490

**Table 4:** Mechanical profile of the PU composites induced with GO.

Sample Code	HS (wt%)	GO (wt%)	*E′* (MPa)	E* (MPa)	D* (1/MPa)	*N* 1 × 10^−4^ (mol/m^3^)	Sample Code	HS (wt%)	GO (wt)%	*E′* (MPa)	E* (MPa)	D* (1/MPa)	*N* 1 ˟ 10^−4^ (mol/m^3^)
PUB	5.0	--	1.6 ± 0.50	1.41 ± 0.25	0.614 ± 0.01	1.17 ± 0.10	PUB	5.0	--	0.20 ± 0.5	0.206 ± 0.5	0.614 ± 0.01	0.148 ± 0.75
PUM1	10.0	0.5	1.65 ± 0.15	1.63 ± 0.75	0.411 ± 0.01	1.06 ± 0.20	PUH1	10.0	0.5	1.6 ± 0.55	1.63 ± 0.25	0.411 ± 0.02	1.06 ± 0.25
PUM2	15.0	1.0	2.49 ± 0.25	2.43 ± 0.75	0.711 ± 0.02	1.82 ± 0.15	PUH2	15.0	1.0	3.76 ± 0.58	3.72 ± 0.58	0.135 ± 0.01	2.76 ± 0.15
PUM3	20.0	1.5	5.26 ± 1.00	5.24 ± 1.00	0.191 ± 0.001	3.86 ± 0.15	PUH3	20.0	1.5	7.4 ± 0.57	7.39 ± 0.80	0.269 ± 0.01	5.58 ± 0.90
PUM4	25.0	2.0	5.92 ± 1.00	5.8 ± 0.900	0.172 ± 0.01	4.34 ± 0.25	PUH4	25.0	2.0	27.59 ± 0.50	36.2 ± 1.50	0.0143 ± 0.001	0.002 ± 0.005

## Data Availability

The data are available on request.
